# Impact of Fertilization on Antioxidant Activity, Microbiological Quality, and Sensory Attributes of Tomato (*Solanum lycopersicum* L.) Cultivated in Ethiopia's Central Rift Valley

**DOI:** 10.1002/fsn3.70609

**Published:** 2025-07-18

**Authors:** Kidist Teshome, Shimeles Aklilu, Ashagrie Zewdu

**Affiliations:** ^1^ Department of Horticulture College of Agriculture and Natural Resource Science, Debre Berhan University Debre Berhan Ethiopia; ^2^ Department of Food Science and Nutrition Collage of Natural and Computational Science, Addis Ababa University Addis Ababa Ethiopia; ^3^ Department of Horticulture Melkassa Agricultural Research Center Melkassa Ethiopia

**Keywords:** bioactive compounds, chemical fertilization, compost, number of days stored, varieties

## Abstract

Tomatoes, known for their antioxidant capacity, are mostly recommended by health professionals. Despite their nutritional significance, progress in enhancing their nutritional qualities and safety has been limited. This study evaluated the effects of compost‐based (organic) and chemical‐based (conventional) fertilization on the antioxidant activity, microbiological quality, and sensory attributes of four commonly cultivated tomato varieties (*Melka Salsa*, *Melka Shola*, *Gelilema*, and *Galilea*) in Ethiopia's Central Rift Valley. Compost fertilization significantly increased vitamin C, flavonoids, total phenolics, DPPH radical scavenging activity, and FRAP antioxidant capacity. However, chemical fertilization primarily enhanced ß–carotene and lycopene content across both growing seasons. Microbiological analysis revealed that the highest total plate count (TPC) and coliform bacteria levels were observed in the unfertilized treatment, whereas no *Escherichia coli* was detected in any of the samples. Sensory evaluation indicated no significant differences in tomato quality among the treatments. Overall, these findings suggest that compost fertilization can enhance the production of bioactive compounds and improve the antioxidant activity of tomatoes, particularly in the Gelilema and Galilea varieties.

## Graphical abstract

Compost fertilization significantly increased vitamin C, flavonoids, total phenolics, DPPH radical scavenging activity, and FRAP antioxidant capacity. However, chemical fertilization enhanced beta‐carotene and lycopene content across both growing seasons. Both organic and conventional farming practices pose no significant microbiological risks. Overall, this finding suggests that compost fertilization can enhance the production of bioactive compounds and improve the antioxidant activity of tomatoes, particularly in the Gelilema and Galilea varieties.

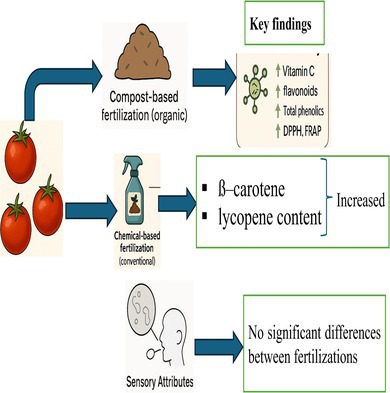

## 1 Introduction

Tomato ( *Solanum lycopersicum* L.) is one of the most widely consumed vegetables globally and ranks as the second most important vegetable crop in terms of production (Pinela et al. 2012). In 2023, global tomato production reached 192.3 million tons (FAO 2024). However, Ethiopia's average tomato yield remains below the global average (Central Statistical Agency (CSA) 2020). Despite this, tomatoes are highly valued for their rich nutritional profile and health benefits. They contain essential bioactive compounds, including minerals, carotenoids (such as lycopene and β‐carotene), vitamins, flavonoids, and polyphenols (De Sio et al. 2021; Ayuso‐Yuste et al. 2022). Lycopene, a potent antioxidant, plays a crucial role in protecting cells from oxidative damage (Dasgupta and Klein 2014) and may contribute to the prevention of certain cancers. The antioxidant levels in tomatoes are influenced by various factors, including the ripening stage, cultivation practices, and climatic conditions (Aldrich et al. 2010). Due to their high lycopene content and the presence of other bioactive compounds, tomatoes are widely recommended as part of a healthy diet (Naeem et al. 2020). The nutritional quality, safety, and consumer appeal of tomatoes are largely affected by cultivation methods, treatments, and varietal differences (Roșca et al. 2023). Fertilization plays a critical role in enhancing the nutritional value and overall quality of crops. While chemical fertilizers effectively increase yields, they are associated with environmental degradation and potential health risks. In contrast, organic fertilization offers a more sustainable alternative (Naeem et al. 2020). Organic fertilizers derived from plant, animal, or microbial sources (such as compost, manure, and biofertilizers) improve soil structure, enhance microbial activity, release nutrients gradually, and minimize nutrient loss. Organic farming practices prioritize ecological balance, promoting sustainable agricultural systems (Gonzalez‐Cebrino et al. 2011).

Organically grown plants are often perceived as safer and richer in antioxidants compared to conventionally grown ones (Czech et al. 2022). While reduced nitrogen availability in organic farming has been suggested to contribute to increased antioxidant capacity, some researchers argue that the overall stress experienced by organically grown crops plays a more significant role in shaping their quality profile (De Pascale et al. 2016). The organic food industry promotes the belief that organic products are healthier, tastier, and more environmentally sustainable. Beyond the final product itself, the value of organic produce extends to the entire production process, emphasizing sustainability and ecological balance (Araujo and Telhado 2015). As a result, consumer demand for organic produce has increased, with many believing that organic farming enhances polyphenolic content and antioxidant capacity in tomatoes (Salama et al. 2015; Aina et al. 2019). However, the use of animal manure as fertilizer raises concerns regarding contamination by pathogenic microorganisms. Despite this, limited research has been conducted on the microbial safety of organically produced foods (Maffei et al. 2016). Although the nutritional quality of vegetables is crucial for human health, little progress has been made in enhancing the nutrient content of tomatoes. This is largely because growers prioritize traits such as disease resistance, shelf life, and visual appeal when selecting cultivars (Basak and Guha 2017). Improving the nutritional quality of tomatoes could provide significant health benefits, which can be achieved by selecting high‐value genotypes adapted to local conditions or by optimizing fertilization practices (Stoleru et al. 2020). Several studies have demonstrated that soil fertility significantly influences the nutritional, antioxidant, and sensory properties of tomatoes (Toor et al. 2006; González‐Coria et al. 2022). However, findings on whether organic produce has superior nutritional quality compared to conventionally grown counterparts remain inconsistent, with some studies disputing the claim (Hallmann 2012; Aires 2016).

Additionally, while organic produce is often perceived as safer, foodborne illness outbreaks have been linked to its consumption (Berger et al. 2010; Jung et al. 2014). This means the use of animal manure as fertilizer raises concerns regarding contamination by pathogenic microorganisms. Despite this, limited research has been conducted on the microbial safety of organically produced foods (Maffei et al. 2016). Numerous scientific studies comparing the microbiological quality of organic and conventionally grown produce have yielded conflicting results (Maffei et al. 2016; Kuan et al. 2017; Xavier et al. 2020). Despite the increasing demand for organic tomatoes and their perceived health benefits, research on the relation of fertilization to the microbiological quality of fresh tomatoes remains limited. Therefore, it is essential to investigate the safety variations in tomatoes grown under organic and conventional farming systems. This study examines the impact of different fertilization methods on the antioxidant status, sensory quality, and microbial safety of four tomato varieties adapted to local environmental conditions at the Melkassa Agricultural Research Center. The hypothesis of the study was that a combination of fertilization and tomato varieties significantly affects the antioxidant activity, microbiological quality, and sensory attributes of tomatoes ( *Solanum lycopersicum* L.) cultivated in Ethiopia's central rift valley, leading to variations in these qualities.

## 2 Materials and Methods

### 2.1 Study Area Description

An open‐field experiment was conducted at the Melkassa Agricultural Research Center (MARC) over two growing seasons (2021 and 2022) under irrigation conditions. The research site is located in southeastern Ethiopia at a geographic coordinate of 8°24′ N latitude and 39°12′ E longitude, with an altitude of 1550 m above sea level (m.a.s.l.). The agro‐ecological zone of the area is classified as semi‐arid, with a mean annual rainfall of 763 mm. The mean annual maximum and minimum temperatures are approximately 28.6°C and 13.8°C, respectively. The soil texture in the study area is predominantly loam to clay loam, and the soil is slightly alkaline, with a pH ranging from 7.4 to 7.6.

### 2.2 Chemical Composition of the Soil and Compost

Composted animal manure was sourced from the Debre‐Zeit Alpha Dairy Farm. The compost was derived from a mixture of cow manure and feed waste, with cows allowed to move and rest on the manure until it decomposed into a stable, fine‐textured compost. Soil samples were randomly collected from the experimental field at a depth of 0–20 cm, targeting the root zone. Samples were taken from seven locations across the field, avoiding border areas. The collected samples were thoroughly mixed to form a composite sample and analyzed for texture and chemical composition at the MARC Soil Analysis Laboratory. Before analysis, both soil and compost samples were air‐dried, ground, and sieved. Particle‐size distribution was determined using the pipette method after the removal of carbonates and organic matter. Additional analysis included pH (1:2.5, w/v), electrical conductivity (1:2, w/v) (Liu et al. 1996). Soil total organic carbon was measured according to a previously described method (Falco and Magni 2004). Total nitrogen, available phosphorous, and exchangeable potassium were analyzed as previously described (Liu et al. 1996).

### 2.3 Treatments and Experimental Design

The experiment was conducted using a factorial randomized complete block design (RCBD) with three replications. Four tomato varieties (*Melka Salsa*, *Melka Shola*, *Gelilema*, and *Galilea*) were tested during the first growing season. Based on the best results for certain nutritional parameters, only *Gelilema and Galilea* were selected for evaluation in the second season to assess their quality attributes and determine the effect of the growing season. Fertilization treatments were applied: compost fertilization (organic), chemical fertilization (inorganic), and control (no fertilization).

### 2.4 Tomato Cultivation Practices

Seeds of each tomato variety were sown in 3 m × 1 m nursery beds, with one seedbed allocated per variety. Weed control and mulching were performed as needed; however, no fertilizer or pesticide was applied during the nursery stage. Seedlings germinated within a week and were transplanted 25 days after sowing. Each plot measured 9 m^2^ (3 m × 3 m), with four rows containing 10 plants each. The spacing and row were 30 cm and 1 m, respectively. The plots were separated by 1.5 m, while blocks were spaced apart. The experiment focused on three fertilization treatments.

1. Organic fertilization: Compost was applied at a rate of 10 tons/ha, with half incorporated 3 weeks before transplanting and the remaining half applied immediately after transplanting. Pest and disease control in organic plots was managed using botanical pesticides (neem extracts).
2. Inorganic fertilization: Chemical fertilizers were applied at rates of 150 kg/ha of Urea (46% N) and 200 kg/ha of blended NPS fertilizer (19% N, 38% P_2_O_5_, and 7% S). Half of the urea was applied at transplanting, with the remaining half applied 35 days post‐transplanting. The same fertilizer rates were maintained across both growing seasons. Fungicides and synthetic pesticides were used for pest and disease control in these plots.
3. Control (no fertilization): These plots did not receive any fertilizer or pesticide treatment as a control.

All plots were irrigated after fertilizer application and transplanting. Tomato plants were stacked for structural support. Data collection was conducted from the middle rows of each experimental plot to minimize edge effects.

### 2.5 Harvesting and Sample Collection

Healthy, undamaged 4 kg tomato fruits were harvested at the breaker stage from each treatment plot. Harvesting took place three to 6 days before testing to ensure proper sample preparation. To minimize physical damage, tomatoes were carefully handled and transported under controlled conditions. Upon arrival at the laboratory, the fruits were sorted based on their treatment groups and placed in trays on a flat surface to prevent bruising or deformation.

### 2.6 Sample Preparation and Analysis of Antioxidant Properties

Twelve tomato fruit samples were blended to create a homogeneous mixture for analysis. Antioxidant properties were measured using three parallel test samples to ensure accuracy and reproducibility. The analysis was conducted at the Addis Ababa University Center of Food Science and Nutrition and the Ethiopian Public Health Institute laboratories using standardized protocols.

### 2.7 Determination of Bioactive Compounds

#### 2.7.1 Vitamin C Analysis

Vitamin C content was determined in triplicate following Vinha et al. (2014). Aliquots of the tomato sample were mixed with 0.1 g/L meta‐phosphoric acid, filtered, and combined with 2,6‐dichloroindophenol. Spectrophotometric readings were taken at 515 nm, and results were expressed as milligrams of vitamin C per 100 g of sample.

#### 2.7.2 Lycopene Content Analysis

Lycopene content was determined in triplicate following the method of Fish et al. (2002) with minor modifications. Frozen tomato samples were thawed and homogenized in 1 mL of distilled water. The homogenate was further diluted in a 2:1:1 ratio with 20 mL of n‐hexane, acetone, and ethanol, respectively. The mixture was then centrifuged, and distilled water was added to facilitate phase separation. The lycopene content was measured spectrophotometrically at 503 nm and estimated using the following Equation (1):

(1) $$\text{Lycopene content}\left(\;\frac{\mathrm{\mu g}}{g}\right)=\left(A*V*10^{6}\right)/\left(3450*w*100\right)$$

where *v* is the amount of hexane (mL), W is the weight of the sample (g), A is the absorbance at 503 nm, and 3450 is the molar extinction coefficient.

#### 2.7.3 ß–Carotene Analysis

Extraction was performed following standard procedures described by Ordóñez‐Santos et al. (2011). 2 g of tomato was weighed in a 250‐mL Erlenmeyer flask, and 50 mL of 2:1:1 hexane/acetone/ethanol containing 2.5% 2,6‐di‐tert‐butyl‐4‐methylphenol (BHT) in acetone was added. The flask was covered with aluminum foil, and N2 was injected for approximately 20 s. The flask was then placed in crushed ice and shaken for 10 min, after which 10 mL of distilled water was added and shaking was continued for a further 5 min. A 4 mL sample of the organic (hexane) phase was then taken with a Pasteur pipette and filtered twice through a 0.2‐pore nylon filter, and 20 μL of the filtrate was injected into an HPLC apparatus equipped with a UV–VIS diode array detector. The mobile phase was a 2 mL min^−1^ flow of 67:27:7 (v/v/v) methanol/tetrahydrofuran (THF)/water, and the columns were thermos‐statted at 30°C. The detection was performed at 450 nm.

### 2.8 Extraction Method for Bioactive and Antioxidant Analysis

The extraction procedure followed the method described by Kähkönen et al. (1999). A 10 g sample was extracted using 20 mL of 80% ethanol. The extracts were then combined and adjusted to100 mL. The prepared extracts were stored at −20°C and used within 3 weeks to ensure sample stability and accuracy of analysis.

### 2.9 Evaluation of Bioactive Compounds

#### 2.9.1 Total Phenolic Content (TPC_GAE_ ) Analysis

Total phenolic content was determined using the method described by Surana et al. (2016). Sample extract (1 mg/mL) was mixed with gallic acid standard, Folin–Ciocalteu reagent, and distilled water. The mixture was incubated, followed by sodium carbonate (Na_2_CO_3_) solution being added. After incubation, the absorbance was measured spectrophotometrically at 765 nm. A standard curve was constructed using gallic acid solutions (20–100 mg/L). Results were expressed as milligrams of gallic acid equivalents per gram of fresh weight (mg GAE/g FW). All samples were analyzed in triplicate to ensure accuracy and reproducibility.

#### 2.9.2 Total Flavonoid Content (TFC) Analysis

Total flavonoid content was estimated using a modified method of Nour et al. (2015) Tomato extract (0.5 mg/mL) was diluted 1:10 with ethanol. To this mixture, 10% AlCl_3_, potassium acetate, and ethanol were added. The solution was then incubated for 40 min at 22°C ± 1°C, and absorbance was measured spectrophotometrically at 415 nm. A quercetin standard curve was used for calibration. Results were expressed as milligrams of quercetin equivalents per gram of fresh weight (mg QE/g FW).

### 2.10 Evaluation of the Antioxidant Activity

#### 2.10.1 DPPH Radical‐Scavenging Activity

DPPH radical‐scavenging activity was measured using a modified method of (Brand‐Williams et al. 1995). A 50 μL sample extract was mixed with 2 mL of 0.1 mM DPPH solution and incubated at 37°C for 30 min. The absorbance was then measured spectrophotometrically at 517. Antioxidant capacity was expressed as mmol Trolox equivalents (TE)/100 g of fresh weight (fw). A Trolox standard curve ranging from 100 to 1000 μmol/L was used for calibration.

#### 2.10.2 Ferric Reducing Antioxidant Power (FRAP)

FRAP was determined following the method described by Benzie and Strain (1999). The FRAP reagent, composed of acetate buffer, TPTZ, and FeCl_3_, was mixed with the sample extract and incubated. The intensity of the resulting blue color, proportional to the antioxidant activity, was measured spectrophotometrically at 593 nm. Results were expressed as mmol Trolox equivalents per gram of fresh weight (mmol TE/g FW).

### 2.11 Sensory Analysis

Sensory evaluation was conducted using fresh raw tomatoes at the light‐red ripening stage. A panel of eleven semi‐trained assessors, aged 18–50 years, participated in the evaluation. All panelists were regular consumers of fresh tomatoes and had no known tomato allergies.

Two tomato varieties were selected for sensory analysis based on their nutritional and antioxidant superiority. Panelists assessed the samples for taste, color, aroma, flavor, and overall quality using a structured 9‐point scale (Villanueva 2003). Final scores were calculated as mean values to determine sensory acceptability.

### 2.12 Physical Quality Analyses

The physical quality of fresh raw tomatoes at the breaker stage was assessed under ambient storage conditions of approximately 24°C ± 2°C with a relative humidity of 72% ± 9%. The evaluation was conducted over a period of 18 days to monitor changes in quality attributes.

#### 2.12.1 Physiological Loss of Weight (PLW) Analysis

Physiological loss of weight (PLW) was determined by weighing the fruits at the beginning of storage and at subsequent intervals. The weight loss was expressed as a percentage of the weight loss relative to the initial fresh weight of the fruit and calculated using the method described by Koraddi and Devendrappa (2011).

(2) %PLW=Initial weight−Final Weight/Intial weight*100

#### 2.12.2 Percentage of Fruit Decay (PFD) Analysis

Percentage of fruit decay (PFD) was determined by periodically counting the number of rotten and shriveled tomato fruits. The decay percentage was calculated using the method described by Tolasa et al. (2021), using the following equation:

(3) %PFD=No.of decayed fruits/totalnoof fruits*100

### 2.13 Microbial Analysis

A 10‐g tomato sample was mixed with 90 mL of peptone buffer solution (PBS) to prepare a stock solution, with slight modifications to the ISO standard. The mixture was then homogenized in a sterile stomacher bag for 3 min. Serial dilutions were prepared by transferring 1 mL of the stock solution into 9 mL of PBS. Microbial assessments were conducted as follows:

1. Total plate count (TPC_cfu_): Determined using plate count agar with the pour plate technique, and the plates were incubated at 35°C ± 1°C for 48 h.
2. Yeast and mold counts: Assessed using potato dextrose agar via the spread plate method, with plates incubated at 25°C ± 1°C for 3–5 days.
3.
*Escherichia coli* and coliform counts: Evaluate using Petri film media. One mL of the diluted sample was plated onto labeled Petri films and incubated at 37°C for 24 h (*E. coli*) and 48 h (coliforms). Colony counts were determined using a colony counter and expressed as log CFU/mL of sample.

### 2.14 Statistical Analysis

The results are expressed as mean ± standard deviation/standard error. Differences among the treatments were analyzed using analysis of variance (ANOVA) to assess the impact of fertilization treatment and tomato variety on antioxidant properties, microbial quality, and sensory attributes. Mean comparisons were conducted using Duncan's multiple range test at a 5% significance level (*p* < 0.05). Statistical analyses were performed using SAS 9.22 software (SAS Institute 2010).

## 3 Results

### 3.1 Determination of Bioactive Compounds

#### 3.1.1 Vitamin C Content

A significant effect of fertilization type and tomato variety on vitamin C content was observed (*p* < 0.05). In the first season, the highest vitamin C content (12.57 mg/100 g) was recorded in the V4T3 combination, while the lowest (6.19 mg/100 g) was found in the V4T1 combination (Table 1). In the second season, the highest vitamin C content (20.05 mg/100 g) was observed in the V4T1 combination, whereas the lowest (11.09 mg/100 g) was recorded in the V3T2 combination (Table 2). These findings suggest that compost fertilization positively influenced vitamin C content, particularly in the second growing season.

**TABLE 1:** The combined effects of fertilization treatments and tomato varieties on the antioxidant properties of tomato fruit during the first season.

Treatment combination		ß‐Carotene (mg/100 g)	Vitamin C (mg/100 g)	Lycopene (mg/100 g)	TFC (mg QE g^−1^ fw)	TPC (mg GAE 100 g fw)
Melka Shola (V1)	Compost	1.49 ± 0.03^de^	10.4 ± 0.14^c^	3.141 ± 0.07^h^	19.47 ± 2.92^e^	24.03 ± 0.06^a^
	Chemical fertilizer	1.46 ± 0.03^e^	7.82 ± 0.42^e^	3.74 ± 0.09^fg^	27.03 ± 0.34^ab^	9.59 ± 0.84^g^
	Control	1.48 ± 0.02^de^	8.96 ± 0.21^d^	3.1 ± 0.77^h^	6.06 ± 0.15^h^	19.06 ± 0.06^c^
Melka Salsa (V2)	Compost	1.93 ± 0.19^b^	10.3 ± 0.7^c^	5 ± 0.46^d^	27.46 ± 0.38^a^	8.96 ± 0.4 h
	Chemical fertilizer	1.73 ± o0.26^c^	7.67 ± 0.5^ef^	4.76 ± 0.3^e^	21.95 ± 0.12_d_	20.26 ± 0.14^b^
	Control	1.47 ± 0.05^de^	11.53 ± 0.07^b^	7.44 ± 1.21^a^	17.92 ± 0.09^f^	7.84 ± 0.4^i^
Gelilema (V3)	Compost	1.5 ± 0.09^d^	6.63 ± 0.14^gh^	3.77 ± 0.04^f^	14.99 ± 0.11^g^	17.22 ± 0.11^d^
	Chemical fertilizer	1.97 ± 0.03^a^	7.08 ± 0.5^fg^	6.84 ± 0.35^b^	17.01 ± 0.05^f^	10.1 ± 0.16^g^
	Control	1.73 ± 0.17^c^	10.99 ± 0.42^bc^	6.06 ± 0.23^c^	24.42 ± 0.05^c^	10.75 ± 0.27^f^
Galilea (V4)	Compost	1.48 ± 0.19^de^	6.19 ± 0.35^h^	4.67 ± 0.08^e^	28.22 ± 0.09^a^	20.25 ± 0.26^b^
	Chemical fertilizer	1.49 ± 0.10^de^	11.68 ± 0.84^b^	2.86 ± 0.38^i^	25.12 ± 0.12^c^	8.14 ± 0.06^i^
	Control	1.48 ± 0.1^de^	12.57 ± 0.84^a^	3.64 ± 0.09^g^	25.78 ± 0.07^bc^	12.05 ± 0.4^e^
Variety (Var.)		*	*	*	*	*
Fertilizer (Fert)		*	*	*	*	*
Var*Fert.		*	*	*	*	*

*Note:* Different lower‐case letters in a column denote a significant difference at (*p* < 0.05) according to Duncan's test; the data are presented as the mean values ± standard deviation (SD). * indicates significant difference.

Abbreviations: TFC, Total flavonoid content; TPC, Total phenolic content.

**TABLE 2:** The combined effects of fertilization treatments and tomato varieties on the antioxidant properties of tomato fruit during the second season.

Treatment combination		B‐Carotene (mg/100 g)	Vitamin C (mg/100 g)	Lycopene (mg/100 g)	TFC (mg QE g^−1^ fw)	TPC (mg GAE 100 g fw)
Gelilema (V3)	Compost	1.5 ± 0.09^c^	18.61 ± 0.42^b^	3.83 ± 0.32^b^	19.22 ± 0.22^c^	21.56 ± 0.12^a^
	Chemical fertilizer	1.97 ± 0.03^a^	11.09 ± 0.14^d^	2.75 ± 0.28^c^	17.4 ± 0.45^d^	10.27 ± 0.2^c^
	Control	1.73 ± 0.17^b^	12.52 ± 0.21^c^	1.41 ± 0.30^f^	6.9 ± 0.07^f^	5.71 ± 0.2^e^
Galilea (V4)	Compost	1.48 ± 0.19^c^	20.05 ± 0.63^a^	2.51 ± 0.53^d^	28.07 ± 0.03^a^	19.3 ± 0.11^b^
	Chemical fertilizer	1.49 ± 0.1^c^	19.16 ± 0.91^b^	4.01 ± 0.03^a^	22.13 ± 0.22^b^	10.42 ± 0.17^c^
	Control	1.04 ± 0.02^d^	13.02 ± 0.5^c^	1.62 ± 0.43^e^	10.04 ± 0.31^e^	7.38 ± 0.26^d^
Variety (Var.)		*	*	*	*	*
Fertilizer (Fert)		*	*	*	*	*
Var*Fert.		*	*	*	*	*

*Note:* Different lower‐case letters in a column denote a significant difference at (*p* < 0.05) according to Duncan's test; the data are presented as the mean values ± standard deviation (SD). * indicates significant difference.

Abbreviations: TFC, Total flavonoid content; TPC, Total phenolic content.

#### 3.1.2 Lycopene Content

The study found that both fertilizer type and tomato variety had a significant effect on lycopene content. In the first season, the highest lycopene content (7.44 mg/100 g) was recorded in V2 under no fertilization, while the lowest (2.86 mg/100 g) was observed in V4, also after chemical fertilization (Table 1). In the second season, the highest lycopene content (4.11 mg/100 g) was recorded in V4 after chemical fertilization, whereas the lowest (1.41 mg/100 g) was found in V3 under control (no fertilization) conditions (Table 2). These results indicate that lycopene levels did not consistently increase as the growing season progressed, suggesting that varietal differences and fertilization methods play a more dominant role in determining lycopene content.

#### 3.1.3 ß–Carotene Content

The study found that both fertilizers and tomato variety significantly influenced ß‐content across both growing seasons (*p* < 0.05). The highest ß‐carotene content (1.97 mg/100 g) was recorded in the variety V3 under chemical fertilizer in both seasons (Tables 1 and 2). However, the lowest ß‐carotene content (1.04 mg/100 g) was observed in the variety V4 under no fertilizer application (Table 2). The findings also indicated that ß‐carotene content did not vary significantly between growing seasons. Additionally, the results confirmed that both genetic factors (variety) and fertilization method played a crucial role in determining ß‐carotene levels in tomatoes.

#### 3.1.4 Total Phenolic Content (TPC_GAE_ )

The study found that both fertilizer type and tomato variety had a significant effect on total phenolic content (*p* < 0.05). In the first production season, the highest total phenolic content (24.03 mg GAE/g FW) was recorded in variety V1 under compost fertilization, while the lowest (7.84 mg GAE/g FW) was observed in variety V2 with no fertilization (Table 1). In the second production season, the highest total phenolic content (21.56 mg GAE/g FW) was found in variety V3 under compost fertilization, whereas the lowest (5.71 mg GAE/g FW) was recorded in the same variety (V3) under no fertilization (Table 2).

#### 3.1.5 Total Flavonoid Content

Analysis of variance indicated that the combined effect of fertilizers and tomato varieties had a significant impact on total flavonoid content (*p* < 0.05). In the first production season, the lowest flavonoid content (6.06 mg QE/g FW) was recorded in the V1T3 combination, while the highest (28.22 mg QE/g FW) was observed in the V4T1 combination (Table 1). Similarly, in the second production season, the V4T1 combination maintained the highest flavonoid content (28.07 mg QE/g FW), whereas the V3T3 combination had the lowest (6.9 mg QE/g FW) (Table 2).

### 3.2 Antioxidant Activity

#### 3.2.1 DPPH Radical‐Scavenging Activity

**FIGURE 1:**
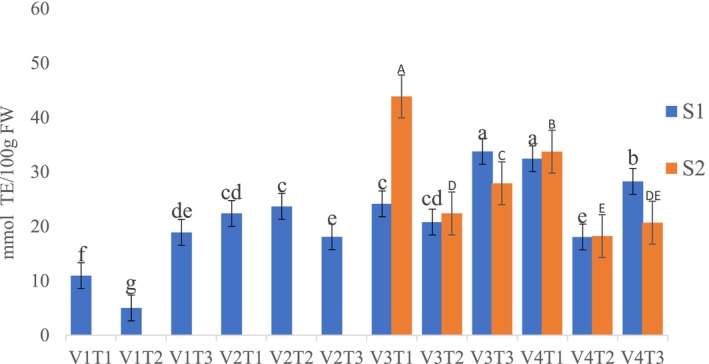
The combined effects of fertilization treatments and tomato varieties on DPPH levels in tomato fruit over two seasons. TE, Trolox Equivalent; FW, Fresh weight; V1—Melka Shola; V2—Melka Salsa; V3—Gelilema; V4—Galilea; T1—Compost; T2—chemical fertilizer; T3—Control (no fertilizer) S1—season one; S2—season two. Different lower‐case and upper‐case letters denote a significant difference at (*p* < 0.05) according to Duncan's test.

The study found that both fertilizer treatment and tomato variety had a significant effect on DPPH across production seasons (*p* < 0.05). In the first season, the highest DPPH radical‐scavenging activities were observed in the V3T3 (33.78 mmol TE/100 g) and V4T1 (32.42 mmol TE/100 g) combinations, with no significant difference between the two (Figure 1). In the second season, the highest DPPH radical‐scavenging activity (43.87 mmol TE/100 g) was recorded in the V3T1 combination, whereas the lowest (18.22 mmol TE/100 g) was observed in the V4T2 combination (Figure 1).

#### 3.2.2 FRAP‐ Ferric Reducing Antioxidant Power

**FIGURE 2:**
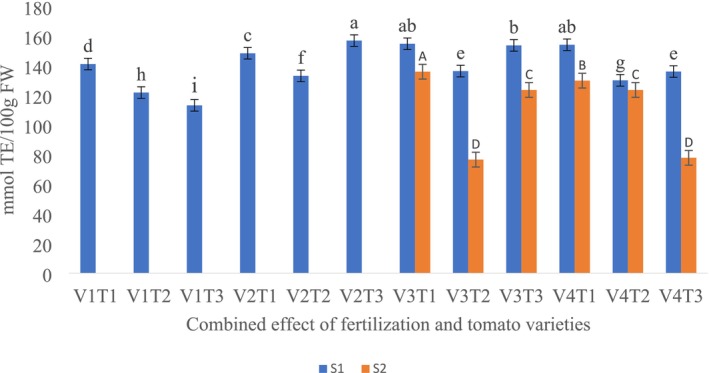
The combined effects of fertilization treatments and tomato varieties on FRAP levels in tomato fruit over two seasons. V1—Melka Shola; V2—Melka Salsa; V3—Gelilema; V4—Galilea; T1—Compost; T2—chemical fertilizer; T3—control (no fertilizer); S1—season one; S2—season two; Different lower‐case and upper‐case letters denote a significant difference at (*p* < 0.05) according to Duncan's test.

The study found that the interaction between fertilizer treatment and tomato variety had a significant effect on FRAP values (*p* < 0.05). In the first season, variety V1 without fertilization had the lowest FRAP value (113.21 mmol TE/100 g), while variety V2, also without fertilization, had the highest FRAP (156 mmol TE/100 g) (Figure 2). In the second season, the highest FRAP value (135.9 mmol TE/100 g) was recorded in variety V3 treated with compost, whereas the lowest (77.81 mmol TE/100 g) was observed in variety V4 under control conditions (no fertilization) (Figure 2).

### 3.3 Sensory Evaluation

**FIGURE 3:**
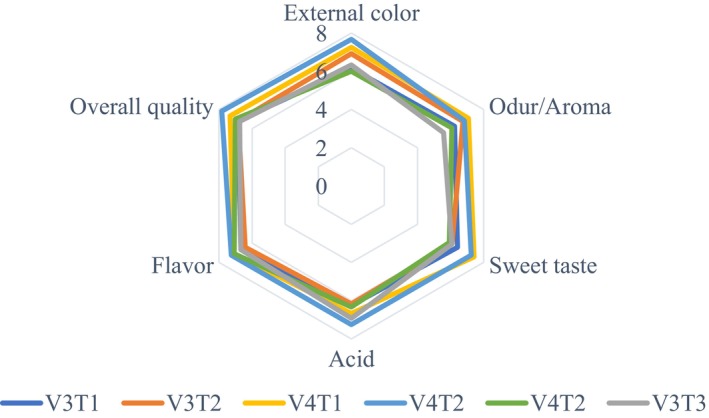
Hue angle measurements for sensorial analysis of the two tomato varieties under different fertilization treatments. V3—Gelilema; V4—alilea; T1—ompost; T2—chemical fertilizer; T3—control.

**FIGURE 4:**
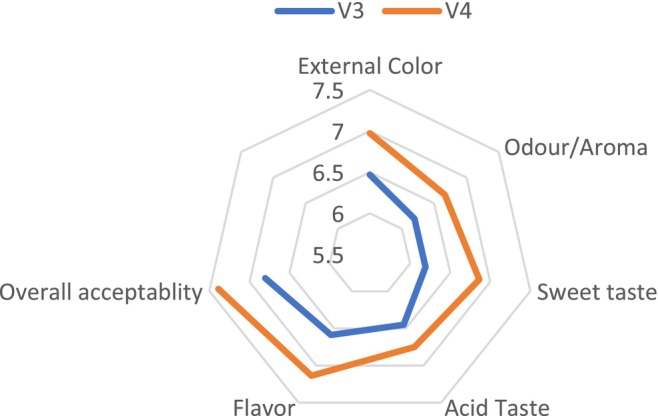
Sensorial evaluation of cumulative effects based on hue angle measurements in two tomato varieties. V3—Gelilema; V4—Galilea.

**FIGURE 5:**
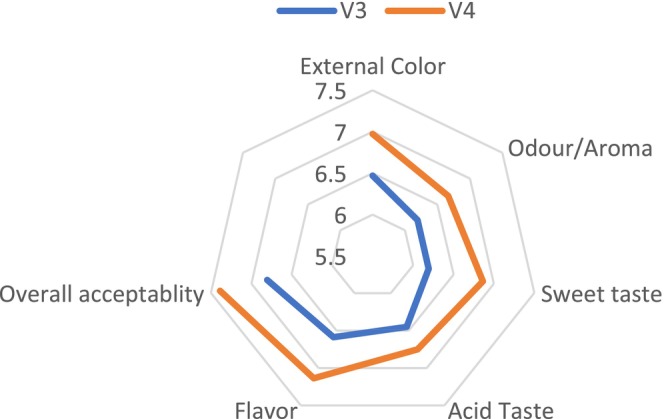
Sensorial evaluation of cumulative effects based on hue angle measurements in two tomato varieties. V3—Gelilema; V4—Galilea.

The study found that neither fertilizer type nor tomato variety had no a significant effect on sensory quality (*p* > 0.05). The analysis of variance revealed that the combination of fertilizers and tomato varieties did not have a significant effect on sensory quality (*p* > 0.05) (Figure 3). No significant differences were observed in external color, aroma, sweetness, acidic taste, flavor, or overall quality among the treatments (Figures 4 and 5). However, the V3T1combination achieved the highest scores for external quality (7.67), overall quality (7.83), flavor (7.08) and acidity (6.92). In terms of aroma (7.08) and sweetness (7.42) and V4T1 was the best combination. These results suggest that compost (organic) fertilization positively influenced sensory attributes, particularly in the V3 and V4 varieties, compared to other fertilization treatments; however, the differences were not statically significant.

### 3.4 Physical Quality Analyses

#### 3.4.1 Physiological Loss of Weight (PLW)

The study revealed that both fertilizer type and tomato variety had a significant effect on weight loss during storage. In the first season, on day eighteen, variety V2 and V3 exhibited the lowest weight loss with compost fertilization. In contrast, the same varieties experienced the highest weight loss on the final day of storage when grown without fertilization. No significant differences in weight loss were observed among treatments until the sixth day of storage (Table 3). In the second season, weight loss increased rapidly in tomatoes grown with chemical fertilization. Compost fertilization resulted in the lowest weight loss, particularly in variety V3. In contrast, chemical fertilization and the control (no fertilization) led to the highest weight loss in both tasted varieties (Table 4).

**TABLE 3:** The interactive effects of fertilization treatments and tomato varieties on the percentage of physiological weight loss (%) of tomato fruits during storage in the first production season.

Physiological loss of weight (PLW) during storage								
Treatment combination		Day 0	Day 3	Day 6	Day 9	Day 12	Day 15	Day 18
Melka Shola (V1)	Compost	0	3.16 ± 2.26	7.69 ± 1.25	18.13 ± 0.65^e^	27.10 ± 1.12^d^	36.84 ± 1.21^f^	51.71 ± 1.07^de^
	Chemical fertilizer	0	3.71 ± 0.70	7.16 ± 1.57	21.19 ± 1.52^de^	33.70 ± 0.64^cd^	45.03 ± 0.21^e^	77.84 ± 5.47^c^
	Control	0	3.21 ± 1.92	9.07 ± 1.58	24.11 ± 1.53^cd^	32.39 ± 0.87^d^	45.17 ± 1.77^e^	73.7 ± 2.29c
Melka Salsa (V2)	Compost	0	5.07 ± 2.81	11.11 ± 7.13	25.24 ± 1.98^c^	32.24 ± 1.23^d^	36.47 ± 3.50^f^	42.52 ± 8.29^f^
	Chemical fertilizer	0	2.669 ± o.18	5.95 ± 0.44	36.00 ± 2.73^b^	47.72 ± 10.12^b^	67.84 ± 0.52^c^	75.73 ± 0.93^c^
	Control	0	4.96 ± 1.52	11.23 ± 2.48	38.18 ± 3.22^b^	73.51 ± 3.65^a^	100^a^	100^a^
Gelilema (V3)	Compost	0	1.92 ± 0.39	4.32 ± 0.25	5.72 ± 1.01^h^	15.62 ± 1.34^e^	22.16 ± 5.37^g^	43.09 ± 1.02^f^
	Chemical fertilizer	0	2.58 ± 0.24	5.60 ± 0.28	14.72 ± 1.63^f^	17.69 ± 1.25^e^	35.22 ± 1.81^f^	46.62 ± 0.9^ef^
	Control	0	2.51 ± 0.89	19 ± 17.87	36.46 ± 1.01^b^	40.12 ± 4.77^c^	55.97 ± 1.32^d^	100^a^
Galilea (V4)	Compost	0	3.31 ± 2.31	15.38 ± 6.82	7.44 ± 1.41^h^	17.33 ± 1.40^e^	21.03 ± 4.05^g^	56.74 ± 1.72^d^
	Chemical fertilizer	0	2.53 ± 0.87	11.35 ± 5.7	45.77 ± 1.84^a^	49.71 ± 7.84^b^	75.97 ± 1.32^b^	84.17 ± 3.03^b^
	Control	0	2.56 ± 0.26	6.57 ± 1.26	10.77 ± 0.91^g^	18.28 ± 0.53^e^	37.5 ± 1.95^f^	74.65 ± 2.65^c^
Variety (Var.)	Ns	Ns	Ns	*	*	*	*	Ns
Fertilizer (Fert)	Ns	*	Ns	Ns	*	*	*	Ns
Var*Fert.	Ns	Ns	Ns	Ns	*	*	*	Ns

*Note:* Different lower‐case letters in a column denote a significant difference at (*p* < 0.05) according to Duncan's test; the data are presented as the mean values ± standard deviation (SD). * indicates significant difference.

**TABLE 4:** The interactive effects of fertilization treatments and tomato varieties on the percentage of physiological weight loss (%) of tomato fruits during storage in the second production season.

Physiological loss of weight (PLW) during storage								
Treatment combination		Day 0	Day 3	Day 6	Day 9	Day 12	Day 15	Day 18
Gelilema (V3)	Compost	0	1.69 ± 0.09	4.31 ± 0.5	6.91 ± 0.83	16.79 ± 1.91^e^	28.25 ± 1.83^d^	38.61 ± 5.45^c^
	Chemical fertilizer	0	2.23 ± 0.22	5.45 ± 0.80	8.31 ± 1.03	51.73 ± 1.15^a^	100^a^	100^a^
	Control	0	1.76 ± 0.21	4.62 ± 0.27	7.80 ± 0.56	21.46 ± 2.02^d^	30.94 ± 0.76^d^	59.44 ± 7.78^b^
Galilea (V4)	Compost	0	1.74 ± 0.08	4.48 ± 0.82	7.65 ± 0.79	12.94 ± 1.63^f^	22.28 ± 1.87^e^	51.99 ± 1.45^b^
	Chemical fertilizer	0	2.11 ± 0.31	5.62 ± 0.94	8.51 ± 1.35	43.88 ± 1.38^b^	70.96 ± 3.42^b^	97.31 ± 4.66^a^
	Control	0	2.03 ± 0.09	4.18 ± 0.80	7.86 ± 0.60	32.77 ± 2.26^c^	61.85 ± 0.93^c^	100^a^
Variety (Var.)		Ns	Ns	Ns	Ns	*	*	*
Fertilizer (Fert)		Ns	Ns	Ns	Ns	Ns	ns	Ns
Var*Fert.		Ns	Ns	Ns	Ns	*	*	*

*Note:* Different lower‐case letters in a column denote a significant difference at (*p* < 0.05) according to Duncan's test; the data are presented as the mean values ± standard deviation (SD). * indicates significant difference.

#### 3.4.2 Percentage of Fruit Decay (PFD)

The interaction between fertilization treatment and tomato variety had a significant effect on PFD (*p* < 0.05) between days 12 and 18 of storage (Table 5). On day 15, the highest PFD (> 50%) was observed across all varieties grown under chemical fertilization and no fertilization. In contrast, the lowest PFD (31.67%) was recorded on day 18 in variety V3, followed by variety V4, which had a PFD of 36.67% under compost fertilization. In the second production season, until day 9, no significant differences in PFD were observed among treatments at that stage (Table 6). However, by day 18, the PFD for all varieties grown under chemical and no fertilization exceeded 50%, though the differences between treatments were significantly different.

**TABLE 5:** The interactive effects of fertilization treatments and tomato varieties on the percentage of decay in tomato fruits during storage in the first season.

Percentage of decay during storage								
Treatment combination		Day0	Day 3	Day 6	Day 9	Day 12	Day 15	Day 18
Melka Shola (V1)	Compost	0	0	6.67 ± 11.55	6.67 ± 11.55	20.00 ± 10.00^c^	30^d^	40^f^
	Chemical fertilizer	0	0	0	13.33 ± 5.77	33.33 ± 5.77^b^	50.00 ± 5.00^c^	80 ± 5^b^
	Control	0	0	10.00 ± 17.32	20.00 ± 5.00	31.67 ± 2.89^b^	51.67 ± 2.89^bcd^	71.67 ± 7.64^cd^
Melka Salsa (V2)	Compost	0	0	0	0	6.67 ± 5.77d	26.67 ± 2.89^d^	41.67 ± 2.89^f^
	Chemical fertilizer	0	0	6.67 ± 5.77	13.33 ± 5.77	36.67 ± 5.77^ab^	68.33 ± 2.89^a^	86.67 ± 2.89^a^
	Control	0	0	10	16.67 ± 5.77	44.67 ± 4.51^a^	56.67 ± 2.89^b^	68.33 ± 2.89^de^
Gelilema (V3)	Compost	0	0	0	10	16.67 ± 2.89^c^	20^e^	31.67 ± 2.89^g^
	Chemical fertilizer	0	0	0	6.67 ± 5.77	31.67 ± 2.89^b^	53.33 ± 2.89^bc^	68.33 ± 2.89^de^
	Control	0	0	3.33 ± 5.77	10.00 ± 10.00	36.67 ± 5.77^ab^	51.67 ± 2.89^bc^	76.67 ± 2.89^bc^
Galilea (V4)	Compost	0	0	0	6.67 ± 5.77	6.67 ± 5.77^d^	11.67 ± 2.89^f^	36.67 ± 2.89^fg^
	Chemical fertilizer	0	0	3.33 ± 5.77	23.33 ± 5.77	33.33 ± 5.77^b^	53.33 ± 5.77^bc^	73.33 ± 2.89^cd^
	Control	0		0	0	30^b^	53.33 ± 2.89^bc^	63.33 ± 2.89^e^
Variety (Var.)		Ns	Ns	Ns	*	*	*	*
Fertilizer (Fert)		Ns	Ns	Ns	Ns	*	*	*
Var*Fert.		Ns	Ns	Ns	Ns	*	*	*

*Note:* Different lower‐case letters in a column denote a significant difference at (*p* < 0.05) according to Duncan's test; the data are presented as the mean values ± standard deviation (SD). * indicates significant difference.

**TABLE 6:** The combined effects of fertilization treatments and tomato varieties on the percentage of decay in tomato fruits during storage in the second season.

Percentage of fruit decay (PFD) during storage								
Treatment combination		Day 0	Day 3	Day 6	Day 9	Day 12	Day 15	Day 18
Gelilema (V3)	Compost	0	0	0	10	13 ± 5.20^c^	20d	31.67 ± 2.89^d^
	Chemical fertilizer	0	0	0	6.67 ± 5.77	41.67 ± 2.89^a^	46.67 ± 5.77^a^	78.33 ± 2.89^a^
	Control	0	0	3.33 ± 5.77	16.67 ± 5.77	26.67 ± 5.77^b^	31.67 ± 2.89	66.67 ± 2.89^b^
Galilea (V4)	Compost	0	0	0	6.67 ± 5.77	6.67 ± 5.77^c^	10^e^	45 ± 5^c^
	Chemical fertilizer	0	0	3.33 ± 5.77	20 ± 17.32	41.67 ± 2.89^a^	48.33 ± 5.77^a^	66.67 ± 2.89^b^
	Control	0	0	0	0	35 ± 5.00^ab^	39.67 ± 0.58^b^	63.33 ± 5.77^b^
Variety (Var.)		Ns	ns	Ns	*	*	*	*
Fertilizer (Fert)		Ns	ns	Ns	*	ns	ns	ns
Var*Fert.		Ns	ns	Ns	*	*	*	*

*Note:* Different lower‐case letters in a column denote a significant difference at (*p* < 0.05) according to Duncan's test; the data are presented as the mean values ± standard deviation (SD). * indicates significant difference.

### 3.5 Microbial Quality

The study found that fertilizer type and tomato variety had a significant effect on the microbial quality of tomato (*p* < 0.05) (Table 7). In the first season, the highest total plate count (TPC_cfu_) 3.54 log cfu/mL was recorded in the variety V2 with no fertilization, while the lowest of 1.15 log cfu/mL was observed in variety V4 under the same treatment. In the second season, the highest TPC_cfu_ of 2.52 log cfu/mL was found for variety V4 with no fertilization. However, TPC_cfu_ was not detected in any variety grown with compost fertilization (Table 6). Similarly, in the first season, the highest coliform bacteria count (2.82 log cfu/mL) was observed in variety V1 with no fertilization, while the lowest (0.65 log cfu/mL) was recorded in variety V2 with chemical fertilization. In contrast, no coliform bacteria were detected in any treatment during the second season. The analysis of variance further revealed that fertilizer treatments and tomato varieties had no significant effect on *E. coli* , yeast, or mold populations (*p* > 0.05) in either season. *E. coli* was not detected in any treatment across both production seasons. Yeast and mold were present in the first season, but their levels did not show significant differences among treatments. In the second season, no yeast or mold was detected across any treatment (Table 7).

**TABLE 7:** The combined effects of fertilization treatments and tomato varieties on the microbial quality of tomato fruit during the first and second seasons.

Treatment combination		TPC (log cfu/mL)		Coliform (log cfu/mL)		*E. coli* (log cfu/mL)		Yeast and mold (log cfu/mL)	
		First season	Second season	First season	Second season	First season	Second season	First season	Second season
Melka Shola (V1)	Compost	2.31 ± 0.33^abcd^	—	1.99 ± 0.30^abc^	—	n.d	—	1.09 ± 1.54	—
	Chemical fertilizer	3 ± 0.02^ab^	—	2.73 ± 0.07^a^	—	n.d	—	2.05 ± 0.21	—
	Control	2.58 ± 0.14^abc^	—	2.82 ± 0.06^a^	—	n.d	—	1.72 ± 0.34	—
Melka Salsa (V2)	Compost	2.35 ± 0.49^abcd^	—	2.23 ± §0.04^ab^	—	n.d	—	2.45 ± 0.21	—
	Chemical fertilizer	2.19 ± 0.16^bcd^	—	0.65 ± 0.92^d^	—	n.d	—	2.87	—
	Control	1.15 ± 1.63^cd^	—	1.69 ± 0.12^abcd^	—	n.d	—	1.48	—
Gelilema (V3)	Compost	3.08 ± 0.01^ab^	n.d	0.95 ± 1.35^cd^	n.d	n.d	n.d	1.78 ± 0.43	n.d
	Chemical fertilizer	1.7^cd^	2.2 ± 0.28^b^	1.30^bcd^	n.d	n.d	n.d	2.67 ± 0.06	n.d
	Control	2.88 ± 0.14^abc^	n.d	2.46 ± 0.02^ab^	n.d	n.d	n.d	2.45 ± 0.21	n.d
Galilea (V4)	Compost	2.59 ± 0.05^abc^	n.d	1.8 ± 0.28^abcd^	n.d	n.d	n.d	2.32 ± 0.09	n.d
	Chemical fertilizer	2.17 ± 0.12^bcd^	n.d	1.8 ± 0.28^abcd^	n.d	n.d	n.d	2.41 ± 0.34	n.d
	Control	3.54^a^	2.52 ± 0.06^a^	2.38 ± 0.05^ab^	n.d	n.d	n.d	2.59 ± 0.08	n.d
Variety (Var.)		Ns	*	Ns	Ns	ns	ns	*	Ns
Fertilizer (Fert)		*	Ns	*	Ns	ns	ns	*	Ns
Var*Fert.		*	*	*	Ns	ns	ns	ns	Ns

*Note:* Different lower‐case letters in a column denote a significant difference at (*p* < 0.05) according to Duncan's test; the data are presented as the means ± standard deviation (SD). —, not studied for second season. * indicates significant difference.

Abbreviations: n.d., not detected; TPC, Total Plate Count.

## 4 Discussion

### 4.1 Total Phenolic and Flavonoid Content

The study revealed that tomatoes grown with compost fertilizer exhibited higher total phenolic content compared to those grown with chemical fertilizers or under no fertilization. These findings align with previous research by Yu et al. (2018), Vélez‐Terreros et al. (2021), and Pradhan and Srijaya (2022) which also reported increased phenolic accumulation in organically cultivated tomato. The accumulation of secondary metabolites, such as flavonoids and phenolic acids, plays a crucial role in plant defense mechanisms and is influenced by various stress factors (Czech et al. 2022), including species type, geographic origin, and cultivation methods (Biondi et al. 2021). Organic soils, which contain higher levels of organic matter, enhance the carbon‐to‐nitrogen ratio, promoting the synthesis of secondary metabolites like flavonoids (Deng et al. 2019). This is because organic fertilizers stimulate the acetate‐shikimate pathway, leading to higher production of flavonoids and phenolic compounds. Additionally, the increased photo‐pathogenic stress in organic farming systems is known to enhance phenolic and flavonoid synthesis, contributing to higher antioxidant activity (Oliveira et al. 2013). Plants grown under organic fertilization often exhibit higher concentrations of these compounds due to lower nitrogen availability, which influences their metabolic pathways (Mitchell et al. 2007). However, our findings contrast with those of de Lima et al. (2024), who reported no significant differences in phenolic and flavonoid content between tomatoes cultivated under organic and conventional farming systems, whether analyzed on a dry or fresh weight basis. This discrepancy may be attributed to differences in soil composition, climate conditions, and fertilization practices, which can influence the accumulation of bioactive compounds.

### 4.2 Vitamin C Content

Fresh tomato typically contains 1.00–63.8 mg/100 g vitamin C (Hallmann 2012; Vélez‐Terreros et al. 2021). Our study results fall within this range, with compost‐fertilized tomatoes exhibiting the highest vitamin C content in comparison to chemical fertilization. These results are consistent with those observed by (El‐Bassel and EI‐Gazzar 2019), Vélez‐Terreros et al. (2021), and Vélez‐Terreros et al. (2024) who reported that tomatoes produced under organic systems frequently had higher contents of vitamin C when compared with those produced conventionally. This trend can be attributed to organic farming practices, which often create mild stress conditions that trigger the accumulation of soluble solids, including vitamin C (Oliveira et al. 2013). Organic fertilizers release nitrogen more gradually than chemical fertilizers, requiring plants to adapt to limited nutrient availability. This nutrient stress may enhance carbohydrate and secondary metabolism (Yu et al. 2018), ultimately stimulating vitamin C production (Worthington 2001). However, contradictory findings have been reported by Barrett et al. (2007) and Ogunleye et al. (2021), suggesting that tomatoes treated with inorganic fertilizers, which provide quick and readily available nitrogen, may also exhibit higher vitamin C content. These discrepancies could be attributed to differences in fertilization regimes, soil composition, climate conditions, and tomato varieties, all of which influence vitamin C synthesis and accumulation.

### 4.3 ß‐Carotene Content

The ß‐carotene content in our study ranged from 1.07 to 1.97 mg/100 g, which is consistent with the findings of Vélez‐Terreros et al. (2021). Tomatoes grown with chemical fertilizers exhibited higher ß‐carotene content in both growing seasons, aligning with the results reported by Aina et al. (2019). While some studies, Oliveira et al. (2013), Vinha et al. (2014), and Rusu et al. (2023) suggest that organic fertilization enhances ß‐carotene level, our findings indicate a stronger correlation between chemical fertilization and increased ß‐carotene accumulation. This may be attributed to the higher phosphorus content in NPK fertilizers, which has been shown to stimulate ß‐carotene synthesis in fruits and vegetables (El‐Baky et al. 2010). These results suggest that nutrient availability, particularly phosphorus, plays a key role in ß‐carotene biosynthesis, which could explain the higher ß‐carotene content observed in chemically fertilized tomatoes compared to those grown under organic conditions.

### 4.4 Lycopene Content

Lycopene is the primary natural antioxidant in tomatoes and is responsible for their red pigmentation. Its concentration can vary depending on several factors, including geographical location, variety, maturity stage, season, and processing methods (Szabo et al. 2018). In this study, lycopene content ranged from 2.8 to 7.4 mg/100 g, aligning with previous research findings (Maruyama et al. 2015; Ochoa‐Velasco et al. 2016; Górecka et al. 2020; Tarihi and Anahtar 2024). Tomatoes grown with chemical fertilizers exhibited the highest lycopene content, while those grown without fertilization had the lowest levels. This trend is consistent with findings reported by Caruso et al. (2019), Uçurum et al. (2019), and Ogunleye et al. (2021) which suggest that chemical fertilization enhances lycopene synthesis. However, Stoleru et al. (2020) and Turhan and Özmen (2021) proposed that organic fertilization can also increase lycopene concentration, attributing this effect to improved chemical and biological activity in organic soils. Organic fertilizers and biofertilizers have been shown to enhance nutrient accumulation in fruits by activating photoinhibition, transport processes, and nitrogen uptake genes (Sharpe et al. 2020).

### 4.5 Antioxidant Activity

The concentration of antioxidants in tomatoes is influenced by both genetic and environmental factors, including variety (genotype), soil conditions, nutrient availability, climate, and pest control practices (Kanabur and Reddy 2014; Vélez‐Terreros et al. 2021). Organic tomatoes have been reported to contain higher levels of polyphenolic compounds, which possess numerous pharmacological properties and act as antioxidants, enhancing the body's defense mechanisms and playing a crucial role in preventing diseases caused by free radicals (Czech et al. 2022). Previous studies by Faller and Fialho (2010) and Kanabur and Reddy (2014) indicate that organic tomatoes contain higher concentrations of phytochemicals, which are strongly associated with greater antioxidant activity. Antioxidant potential is commonly measured using tests such as FRAP (Ferric Reducing Antioxidant Power), which assesses a substance's ability to reduce ferric ions. Our study found that tomatoes fertilized with compost, which contains lower nitrogen levels, exhibited higher FRAP values than those grown under chemical fertilization or no fertilization in both seasons. These findings align with research by El‐Baky et al. (2010), Kanabur and Reddy (2014), Czech et al. (2022), and de Lima et al. (2024) which suggest that organic tomatoes, often limited in nitrogen availability, may accumulate higher FRAP concentrations compared to conventionally grown tomatoes. A similar trend was observed for DPPH radical‐scavenging activity, another widely used measure of antioxidant potential. Studies by Vinha et al. (2014) and de Lima et al. (2024) also found that organic tomatoes exhibit stronger DPPH radical‐scavenging ability than those cultivated under conventional farming practices. These highlight the role of nutrient stress in stimulating antioxidant production, suggesting that organic fertilization methods may enhance the antioxidant capacity of tomatoes by promoting secondary metabolite synthesis.

### 4.6 Physiological Loss in Weight (PLW)

In this study, tomatoes grown with chemical fertilizers exhibited the highest physiological loss in weight (PLW) compared to those grown with organic fertilizers or no fertilization. These findings align with previous research by Muthukumar (2019) and Vélez‐Terreros et al. (2021) which suggest that the absence of essential micronutrients in chemical fertilizers weakens the fruit's cellular structure, leading to increased moisture content and greater PLW. Conversely, studies by Laxmi et al. (2015) and Verma et al. (2020) observed that organic manures help extend shelf life by providing essential micronutrients and reducing moisture content, thereby minimizing weight loss during storage. This suggests that organic fertilization may contribute to better post‐harvest stability in tomatoes compared to chemical fertilization.

### 4.7 Sensory Quality

Although relatively few studies have examined the sensory profiles of different tomato varieties, our findings indicate that there were no significant differences in sensory attributes among fertilization treatments or varieties. This result is consistent with previous research by Kapoulas et al. (2011), Tobin et al. (2013), and Araujo and Telhado (2015), which also found no statistically significant variation in sensory quality due to fertilization methods. However, our study suggests that compost‐grown tomatoes may demonstrate modestly enhanced sensory qualities, though these differences did not reach statistical significance. These highlight that while fertilization type plays a critical role in post‐harvest quality, its impact on sensory perception remains minimal, reinforcing the idea that consumers may not perceive significant taste or texture differences between organically and conventionally grown tomatoes.

### 4.8 Microbiological Quality and Food Safety

The study found no evidence of *E. coli* contamination in any of the fertilization treatments or tomato varieties during both growing seasons. These results align with findings by Wießner et al. (2009), who reported no correlation between the presence of *E. coli* in lettuce and the type of fertilizer used. The use of mature compost and the avoidance of direct application of composted manure to ready‐to‐eat crops during the growing season may have contributed to minimizing the risk of *E. coli* contamination. This approach is consistent with commercial organic production guidelines, which emphasize safe composting practices to prevent microbial contamination. In this study, yeast and mold were detected in the first season but showed no significant differences between treatments. In the second season, no yeast or mold was detected in any of the treatments. These findings agree with research by Vélez‐Terreros et al. (2021) and Castro‐Urbina et al. (2023) which found no significant differences in yeast and mold levels between conventionally and organically grown vegetables. Additionally, the study found that the highest coliform bacteria count was in the no fertilization treatment. However, Mukherjee et al. (2004) reported no significant differences in coliform counts when comparing organic and chemical fertilization in lettuce, cabbage, tomatoes, green peppers, and cucumbers. Overall, the findings suggest that both organic and conventional farming practices pose no significant microbiological risks. These results are consistent with Kuan et al. (2017) who concluded that farming practices do not significantly impact the microbiological safety profiles of fresh produce.

## 5 Conclusion

This study demonstrated notable differences between organic and conventional tomatoes in terms of bioactive compounds, antioxidant activity, and microbial quality. However, sensory attributes did not differ significantly among the different fertilization treatments. Tomatoes grown with compost (organic fertilization) exhibited higher bioactive compound content and stronger antioxidant activity compared to those cultivated using chemical fertilizers, particularly in the V3 and V4 varieties. Sensory evaluations revealed no significant differences between fertilization treatments, indicating that organic and conventionally grown tomatoes offer comparable taste and quality. Additionally, farming practices did not significantly impact the microbiological profiles of fresh tomatoes, confirming that both organic and conventional production methods can ensure microbial safety when proper agricultural practices are followed. These findings suggest that consuming organic tomatoes may be beneficial due to their higher phenolic and flavonoid content and enhanced antioxidant capacity. Overall, the use of compost as a fertilization strategy can increase secondary metabolite production and improve antioxidant properties in tomatoes without compromising microbial safety.

## Author Contributions

**Kidist Teshome:** conceptualization (equal), data curation (equal), formal analysis (lead), investigation (equal), methodology (equal), resources (equal), validation (equal), visualization (equal), writing – original draft (lead). **Shimeles Aklilu:** conceptualization (equal), methodology (equal), resources (equal), supervision (equal), validation (equal). **Ashagrie Zewdu:** conceptualization (equal), data curation (equal), methodology (equal), resources (equal), supervision (equal), validation (equal), visualization (equal), writing – review and editing (lead).

## Ethics Statement

This study was approved by the Institutional Review Board of Addis Ababa University.

## Consent

Written informed consent was obtained from all study participants.

## Conflicts of Interest

The authors declare no conflicts of interest.

## Data Availability

The data will be made available upon request.
